# Interleukin 17A: A Potential Target for Its Plausible Roles in the Pathogenesis of Idiopathic Epistaxis

**DOI:** 10.22038/IJORL.2021.51208.2731

**Published:** 2022-01

**Authors:** Reza Nosratabadi, Ali Golshiri-Isfahani, Soheila Rahmanzadeh-Shahi, Gholamreza Asadikaram, Mohammad Kazemi Arababadi

**Affiliations:** 1 *Department of Medical Immunology, Afzalipour Faculty of Medicine, Kerman University of Medical Sciences, Kerman, Iran.*; 2 *Department of Otolaryngology, Faculty of Medicine, Rafsanjan University of Medical Sciences, Rafsanjan, Iran.*; 3 *Immunology of Infectious Diseases Research Center, Research Institute of Basic Medical Sciences, Rafsanjan University of Medical Sciences, Rafsanjan, Iran.*; 4 *Neuroscience Research Center, Institute of Neuropharmacology, and Department of Biochemistry, Afzalipour Faculty of Medicine, Kerman University of Medical Sciences, Kerman, Iran.*; 5 *Department of Laboratory Sciences, Faculty of Paramedicine, Rafsanjan University of Medical Sciences, Rafsanjan, Iran.*

**Keywords:** Epistaxis, Inflammation, IL-17A

## Abstract

**Introduction:**

It has been reported that inflammation may be a potential risk factor for the progression of epistaxis. Due to the major roles played by Th17 in the induction of inflammation, the present study aimed to assess the serum levels of Interleukin 17A (IL-17A) and IL-23, as the most important cytokines in the Th17 pathway, as well as IFN-, IL-4, and IL-10 serum levels, as regulatory cytokines for Th17 cells in patients with idiopathic epistaxis.

**Materials and Methods:**

The serum levels of IL-4, IL-10, IL-17A, IL-23, and IFN- were evaluated in 90 patients with idiopathic epistaxis and 90 healthy controls using enzyme-linked immunosorbent assay (ELISA) technique.

**Results:**

The obtained results pointed out that the serum levels of IL-17A and IL-10, but not IL-4 and IL-23, were significantly up-regulated, and IFN- serum levels were significantly down-regulated in patients with idiopathic epistaxis. Furthermore, female patients with epistaxis had higher IL-10 serum levels.

**Conclusions:**

As evidenced by the results of the present study, IL-17A is the main cytokine which participates in the pathogenesis of idiopathic epistaxis; moreover, in association with IL-10, it can be regarded as the suppressor of IFN- in patients.

## Introduction

Epistaxis is a disorder associated with spontaneous bleeding from the nose and is highly prevalent in tropical areas (1). The main etiology and mechanisms of epistaxis are yet to be cleared; therefore, epistaxis is regarded as an idiopathic disorder in some situations. Although some reports demonstrated that epistaxis may occur in patients suffering from allergy (2), the roles of Th1, Th2, and Th17 immunity needs to be clarified in patients with epistaxis. Chronic inflammation may be a potential risk factor for the progression of epistaxis (3); therefore, the T helper lineages may critically participate in the pathogenesis of epistaxis. Our previous investigation revealed that Interleukin 6 (IL-6) and transforming growth factor (TGF)-β are up-regulated in patients with epistaxis. 

It has been reported that IL-6 and TGF-β are the most important cytokines for the development of Th17 lineage (4). Therefore, we have hypothesized that Th17 may significantly participate in the pathogenesis of epistaxis. Interleukin 17A (IL-17A) is the most important cytokine which is produced by the Th17 lineage. Moreover, IL-23 is the main cytokine for the maintenance of the cells (5); therefore, this research project was designed to explore IL-17A and IL-23 serum levels in patients with epistaxis, in comparison with healthy controls. In addition, previous investigations reported that IFN-, as the main cytokine produced by Th1 cells, and IL-4, which is the most important cytokine of Th2 cells, and also IL-10, as the crucial anti-inflammatory cytokine, negatively regulate the development of Th17 lineage(6). Therefore, the IFN-, IL-4, and IL-10 serum levels were also determined in patients with epistaxis in comparison with healthy controls. 

Furthermore, due to the fact that IL-8, IL-6, TGF-β, TNF-α, endothelin, and IgE serum levels have been evaluated in these patients in our previous study (7), another aim of the present research was to evaluate the correlation of IL-17A, IL-23, IFN-, IL-4 and IL-10 serum levels with IL-8, IL-6, TGF-β, TNF-α, endothelin, and IgE serum levels. Furthermore, the IL-17A, IL-23, IFN-, IL-4, and IL-10 serum levels were also compared according to gender, antihistamine receiving, nose picking, bleeding of the gums, smoking, familial history of epistaxis, and clinical presentations similar to allergies. 

## Materials and Methods

***Subjects***

The present study was performed on 90 patients with epistaxis and 90 healthy controls who were matched for gender, age (40±2.27 for patients and 37.31±2.4 years for controls), and smoking status (10 and 8 participants in patients and controls, respectively) in 2018. The main bias was the gender distribution since most participants were female and it may have affected the results. Therefore, we selected the same pattern in the controls. Accordingly, this study recruited 90 patients with idiopathic epistaxis (32 males and 58 females) and 90 healthy controls (28 males and 62 females). The patients and corresponding control subjects were selected from the individuals who have been referred to the Moradi-Hospital, Rafsanjan, Iran.

The factors which may affect epistaxis and cytokine serum levels were considered the exclusion criteria, including alcohol consumptions, infectious diseases, administrating immunosuppressive drugs, hypertension, hematologic disorders (hemostasis and anticoagulant medications (e.g. warfarin), or antiplatelet drugs (e.g. aspirin) treated patients), tumors, collagen diseases, hemophilia, nose trauma, and systematic bleeding history. An expert specialist physician diagnosed the existence/lack of idiopathic epistaxis and also allergy due to the medical history, clinical symptoms, and physical examinations. 

The participants completed the questionnaire (7) and consent forms. The questionnaire requested demographic data and several relevant information, such as the history of smoking, gum health, past or current nose picking, and epistaxis history, and contained questions to include or exclude from the study, as listed earlier herein. After the selection of the patients and healthy controls, the sera were obtained and the serum levels of IL-17A, IL-23, IFN-, IL-4, and IL-10 were evaluated to compare the patients and healthy controls. The project protocol was approved by the Ethical Committee of Rafsanjan University of Medical Sciences (code: IR. RUMS. 1396.143). 

***Cytokine Assay***

The serum levels of IL-17A, IL-23, IFN-, IL-4, and IL-10 were evaluated using a commercial kit from the Karmania Pars Gene Company, Kerman, Iran, according to the manufacturer’s instructions. In brief, the standards and samples were added to the ELISA vials and incubated for 1 h. Following that, the vials were washed three times and conjugated; subsequently, the antibody was added, and they were washed after 1 hour of incubation. Thereafter, HRP-avidin conjugation was added and after 30 min of incubation and washing, the substrate was added and incubated for 15 min. The reaction was stopped using a stopping solution, and the optical density (OD) was measured at 450 nm.

***Statistical analysis***

Due to the normal distribution of raw data, the analysis of data was calculated using parametric tests; therefore, the student t-test and Pearson's correlation coefficient (r) test were employed for the analysis of the data, and the results were presented as mean±standard error (SE). A p-value of ≤0.05 was considered statistically significant.

## Results

**Fig 1:**
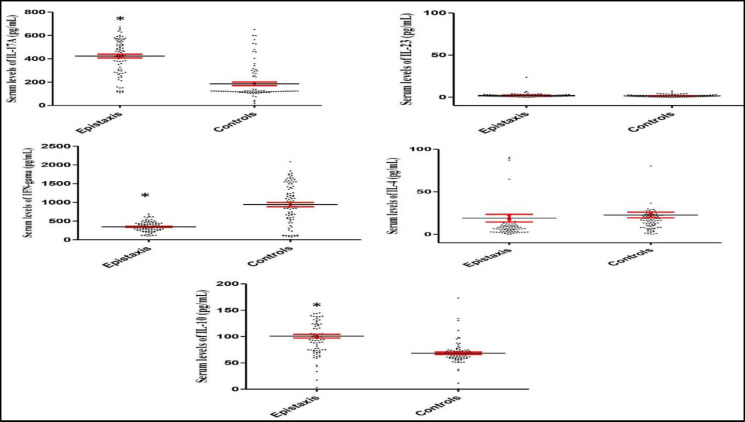
IL-17A, IL-23, IFN- , IL-4, and IL-10 serum levels in the epistaxis patients in comparison to controls. The graphs show that IL-17A and IL-10, but not IL-4 and IL-23, serum levels were significantly up-regulated and IFN-  serum levels were significantly down-regulated in the idiopathic epistaxis patients. *P< 0.001

The results demonstrated that IL-17A serum levels significantly increased (P<0.001; Figure 1) in patients with epistaxis (423.47±16.40 pg/mL), as compared to those in healthy controls (185.88±15.07 pg/mL). Moreover, IL-10 serum levels significantly increased (P<0.001; Figure 1) in patients with epistaxis (100.67±3.56 pg/mL), compared to those obtained in healthy controls (68.33 ± 2.24 pg/mL). However, IFN- serum levels significantly decreased (P<0.001; Figure 1) in patients with epistaxis (346.56±14.29 pg/mL), when compared to healthy controls (938.52±58.28 pg/mL). The statistical analysis showed that IL-4 (P= 0.345) and IL-23 (P=0.198) did not significantly change in the patients, compared to the controls (Figure 1).

**Fig 2:**
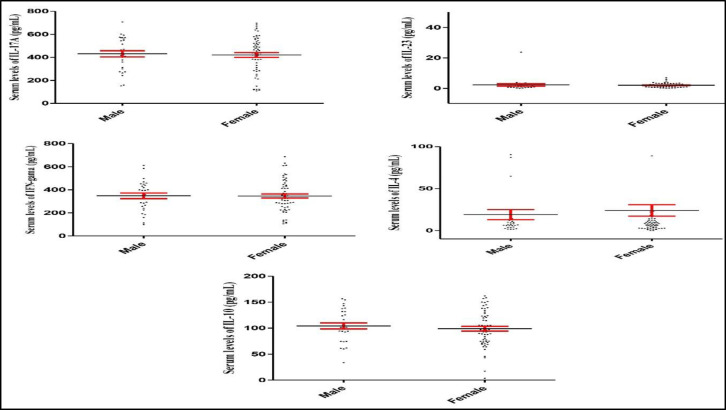
IL-17A, IL-23, IFN- , IL-4, and IL-10 serum levels in the male and female epistaxis patients. The graphs illustrate that IL-17A, IL-23, IFN- , IL-4, and IL-10 serum levels did not alter in female when compared to male patients

Furthermore, IL-17A (P= 0.760), IL-23 (P=0.658), IFN- (P=0.961), IL-4 (P=0.370), and IL-10 (P=0.487) serum levels did not change in female and male patients with epistaxis (Figure 2). 

**Fig 3:**
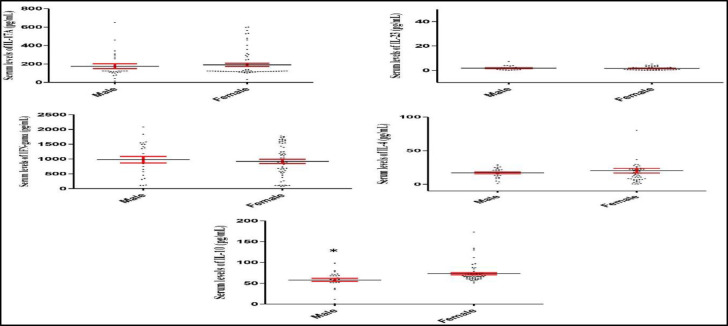
IL-17A, IL-23, IFN- , IL-4, and IL-10 serum levels in the male and female healthy controls. The graphs demonstrate that IL-10 serum levels significantly increased in female controls, when compared to male healthy individuals. The IL-17A, IL-23, IFN- , and IL-4 serum levels did not alter in female when compared to male controls. *P=0.002

The results revealed that IL-10 serum levels significantly increased in females, in comparison with those in male healthy controls (P=0.002; Figure 3). On the other hand, IL-17A (P=0.636), IL-23 (P=0.444), IFN- (P=0.642), and IL-4 (P=0.233) serum levels did not differ in male and female controls. 

**Fig 5:**
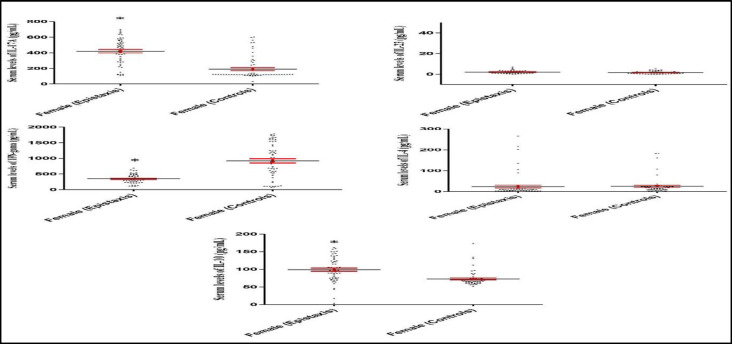
IL-17A, IL-23, IFN- , IL-4, and IL-10 serum levels in the female epistaxis patients versus female healthy controls. Data analysis revealed that IL-17A and IL-10 serum levels significantly increased and IFN-  decreased in female epistaxis patients when compared to female healthy controls, respectively.*P<0.001

The statistical analysis showed that IL-17A and IL-10 serum levels significantly increased and IFN- decreased in both male and female patients with epistaxis, as compared to those in male and female healthy controls, respectively (P<0.001 for all; figure 4 and 5). 

*****Fig 4:**
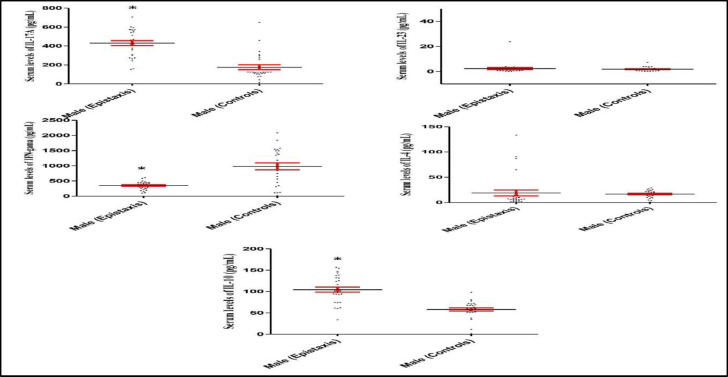
IL-17A, IL-23, IFN- , IL-4 and IL-10 serum levels in male epistaxis patients versus male healthy controls. Data analysis revealed that IL-17A and IL-10 serum levels significantly increased and IFN-  decreased in male epistaxis patients when compared to male healthy controls, respectively. *P<0.001

**Fig 6:**
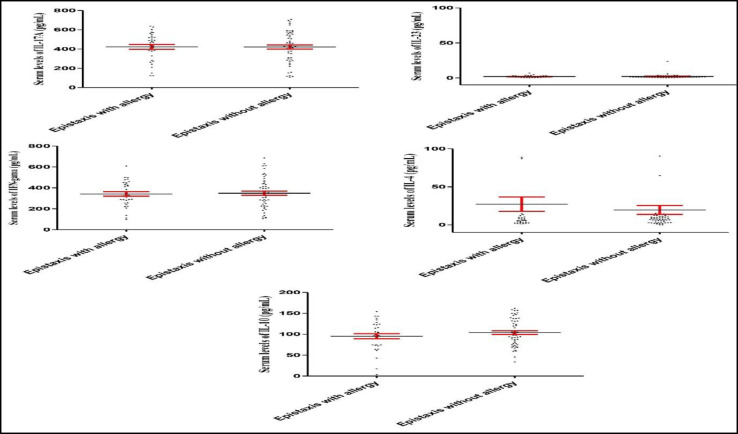
IL-17A, IL-23, IFN- , IL-4, and IL-10 serum levels in the epistaxis patients with and without clinical allergies. There were no significant differences between the epistaxis patients with and without clinical allergies regarding the IL-17A, IL-23, IFN- , IL-4, and IL-10 serum levels

**Fig 7:**
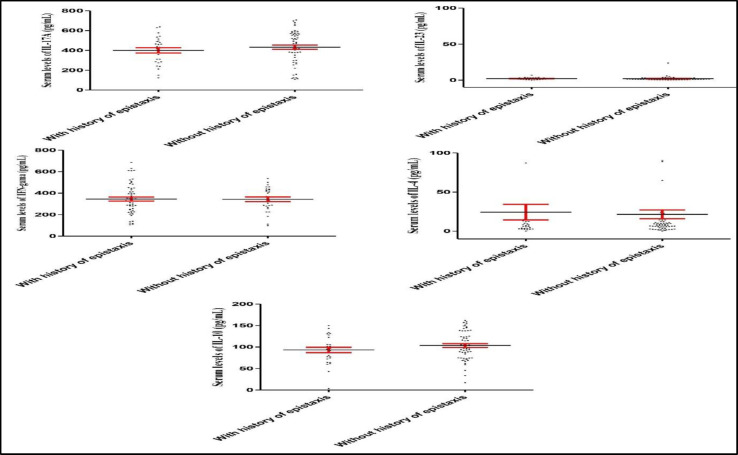
IL-17A, IL-23, IFN- , IL-4, and IL-10 serum levels in the epistaxis patients with and without a familial history of epistaxis. IL-17A, IL-23, IFN- , IL-4 and IL-10 serum levels did not change in the epistaxis patients with and without familial epistaxis history

There were no significant differences between epistaxis patients who suffered from allergy regarding IL-17A (P=0.969), IL-23 (P=0.777), IFN- (P=0.811), IL-4 (P=0.571), and IL-10 (P=0.238) serum levels, when compared to those without allergy (Figure 6). Nonetheless, IL-17A (P=0.325), IL-23 (P=0.930), IFN- (P=0.860), IL-4 (P=0.313), and IL-10 (P=0.158) serum levels did not change in epistaxis patients with and without a familial history of epistaxis (Figure 7). As illustrated by Table 1, although the statistical analysis revealed some poor correlations between the cytokines, there was a significant positive correlation between IL-17A and IL-10. Moreover, a moderate positive correlation was detected between endothelin-1 and IL-10, as well as IFN- and IL-10.

**Table1:** Correlation among variables in the epistaxis

		IFN-	IL-17A	IL-4	IL-10	IL-23
IL-6	Pearson Correlation	-0.065	-0.095	0.068	-0.164	-0.223
	P-value	0.547	0.379	0.523	0.126	0.036
IL-8	Pearson Correlation	0.037	-0.187	-0.062	-0.072	0.138
	P-value	0.729	0.082	0.564	0.508	0.198
TNF-α	Pearson Correlation	0.044	-0.042	-0.090	0.031	-0.011
	P-value	0.682	0.699	0.401	0.775	0.920
TGF-β	Pearson Correlation	0.009	-0.219	-0.010	-0.277	-0.042
	P-value	0.935	0.040	0.924	0.009	0.695
IgE	Pearson Correlation	-0.114	-0.027	-0.102	-0.109	-0.095
	P-value	0.290	0.806	0.337	0.311	0.380
Endothelin-1	Pearson Correlation	0.139	0.289	-0.049	0.342	-0.077
	P-value	0.198	0.006	0.647	0.001	0.478
IFN-	Pearson Correlation	1	0.217	0.135	0.322	-0.062
	P-value		0.042	0.209	0.002	0.564
IL-17A	Pearson Correlation	0.217^*^	1	-0.064	0.730	-0.163
	P-value	0.042		0.555	0.000	0.128
IL-4	Pearson Correlation	0.135	-0.064	1	-0.038	0.026
	P-value	0.209	0.555		0.723	0.811
IL-10	Pearson Correlation	0.322	0.730	-0.038	1	-0.084
	P-value	0.002	0.000	0.723		0.436
IL-23	Pearson Correlation	-0.062	-0.163	0.026	-0.084	1
	P-value	0.564	0.128	0.811	0.436	

Although the statistical analysis revealed some poor correlations between the cytokines, there was a significant positive correlation between IL-17A and IL-10. Moreover, a moderate positive correlation was detected between endothelin-1 and IL-10, as well as IFN- and IL-10.

## Discussion

In the present study, IL-17A, IL-23, IFN-γ, IL-4, and IL-10 serum levels were compared between patients with idiopathic epistaxis and normal controls. The results pointed out that IL-17A and IL-10 serum levels were increased in patients with idiopathic epistaxis, as compared to those in the controls. 

The IL-17A is an inflammatory cytokine produced by several cells, including Th17 lymphocytes (8). IL-17A plays key roles in the pathogenesis of pro-inflammatory-based diseases and also is associated with the fibrosis of human tissues (9). Due to the fact that the etiology of idiopathic epistaxis needs to be cleared, it appears that our assessment of the cytokine status of the disease can elucidate some features of idiopathic epistaxis. As evidenced by the results of the current study, IL-17 can be considered an important target for investigations regarding the etiology of idiopathic epistaxis. In parallel with the findings, our previous investigation revealed that IL-6 and TGF-β serum levels were higher in patients with epistaxis who were evaluated in this study (7). 

Since IL-6 and TGF-β contribute greatly to the development of Th17 lineage (4), the results of the present research confirmed the findings of the previous study and revealed that the up-regulation of IL-6 and TGF-β is associated with increased IL-17A serum levels. Therefore, it appears that Th17 lymphocytes are the main cells which participate in the etiology of idiopathic epistaxis. In addition, it has been demonstrated that Th17 lymphocytes are the main cells participating in the pathogenesis of nasal polyps as a major cause of epistaxis (10). The relationship of IL-17 and IL-10 with nasal polyp has also been reported. Atopic and non-atopic patients with nasal polyp demonstrated that the level of these cytokines was increased in both groups, as compared to that in the controls (11).

The results also indicated that IL-10 serum levels were higher in patients with epistaxis, as compared to those in healthy controls. Furthermore, in other bleeding disorders, such as hemophilia A (HA), the finding suggested that the level of IL-10 increased in HA patients, in comparison with that in the control group (12). Moreover, the levels of IL-6 and IL-10 were higher in patients with intraparenchymal hemorrhage, compared to those in the control group (13). Controversial results have been reported regarding the level of IL-10 in chronic subdural hematoma (CSDH) fluid. For instance, in one study, the level of IL-10 was lower in CSDH fluid than serum, whereas in some other studies, the level was high when comparing CSDH fluid with serum (14). 

On the other hand, it has been documented that IL-10, as a potential anti-inflammatory cytokine, has a major role to play in the inhibition of inflammation within human tissues (15). Due to the increased production of IL-10 in patients with epistaxis, it may be concluded that the up-regulation of IL-10 is a normal immune response to increased expression of IL-17A, performing regulatory functions to modulate tissue damage roles of IL-17A. In line with the hypothesis, the data analysis in Table 1 shows that there was a significant positive correlation between IL-17A and IL-10 serum levels. Moreover, the results pointed out that there were moderate positive correlations between endothelin-1 and IL-10, as well as IFN- and IL-10. Endothelin-1 and IFN- are the important pro-inflammatory cytokines; therefore, their positive correlation with IL-10 serum levels confirmed the roles played by inflammatory molecules in the induction of IL-10 expression.

In addition, the results showed that IFN- serum levels significantly decreased in patients with epistaxis, in comparison with those in controls. The IFN- is a cytokine produced by Th1 lymphocytes (16). It has been reported that there is a negative feedback between IFN- and IL-17A (17). 

In another word, the activation of Th17 cells is associated with the down-regulation of Th1 and Th2 lymphocyte-related genes. Due to the increased IL-17A serum levels, it can be concluded that the up-regulation of cytokine leads to the down-regulation of IFN-, as Th1 cytokine. The down-regulation of IFN- bolds the roles played by IL-17A in the pathogenesis of idiopathic epistaxis. Nevertheless, more investigations by intracellular staining of the T helper lineage can be of great help in determining the activities of Th1 and Th17 cells in patients with epistaxis. 

Data analysis in the male and female patients with epistaxis, in comparison with male and female controls, also confirmed the results and revealed that the serum levels of IL-17A and IL-10, as well as IFN-, had the same patterns in all patients. However, IL-10 serum levels were higher in female controls, in comparison with males. Female sex hormones are the important molecules which shift the Th1/Th2 balance towards Th2 cells (18). 

Moreover, IL-10 is produced by several cells, including Th2 lymphocytes (19). Consequently, it appears that IL-10 was produced in female controls due to various female sex hormones in females, in comparison with controls. Nonetheless, no differences were observed between female and male patients with epistaxis regarding IL-10 serum levels; therefore, it appears that female sex hormones were unable to up-regulate IL-10 in female patients with epistaxis. 

Furthermore, Immunoglobulin E (IgE)  serum levels was not correlated with IL-17A, IL-23, IFN-, IL-4, and IL-10 serum levels. Moreover, there were no differences between epistaxis patients with and without a familial history of epistaxis, as well as with and without allergy. It appears that allergy cannot be considered a risk factor for alteration in the expression of cytokines in the patients. 

On the other hand, a study by Murray et al. on children with allergic rhinitis showed that allergy was associated with epistaxis (20). Therefore, allergy can seemingly lead to the induction of epistaxis; nonetheless, in Iranian patients with idiopathic epistaxis, allergy was not associated with alteration in the expression of cytokines. 

## Conclusion

As evidenced by the results of the present study, IL-17A is the main cytokine that participates in the pathogenesis of idiopathic epistaxis; moreover, in association with IL-10, it can be regarded as the suppressor of IFN- in patients with epistaxis. 
